# Differences between Four Skinfold Calipers in the Assessment of Adipose Tissue in Young Adult Healthy Population

**DOI:** 10.3390/nu14102085

**Published:** 2022-05-16

**Authors:** Francisco Esparza-Ros, Ana Catarina Moreira, Raquel Vaquero-Cristóbal, Carlos Barrigas, Mario Albaladejo-Saura, Filomena Vieira

**Affiliations:** 1 International Kinanthropometry, Universidad Católica de Murcia, 30107 Murcia, Spain; fesparza@ucam.edu (F.E.-R.); mdalbaladejosaura@ucam.edu (M.A.-S.); 2 ESTeSL—Escola Superior de Tecnologia da Saúde de Lisboa, Instituto Politécnico de Lisboa, H&TRC—Health & Technology Research Center, 1990-096 Lisboa, Portugal; ana.moreira@estesl.ipl.pt; 3 Faculty of Sport Sciences, Universidad Católica de Murcia, 30107 Murcia, Spain; 4 Instituto de Estudos Interculturais e Transdisciplinares, 2805-059 Almada, Portugal; cbarrigas@fmh.ulisboa.pt; 5 Laboratory of Biomechanics and Functional Morphology, Interdisciplinary Centre for the Study of Human Performance (CIPER), Faculty of Human Kinetics (FMH), University of Lisbon, 1495-751 Cruz Quebrada, Portugal; fvieira@fmh.ulisboa.pt

**Keywords:** anthropometry, body composition, fat mass, health

## Abstract

Background: The aim of this study was to analyze the validity of four different skinfold calipers, as well as to establish the differences between them in a healthy young adult population. Methods: The present study followed a cross-sectional design, including 138 participants, with 69 males (21.46 ± 2.52 years) and 69 females (22.19 ± 2.85 years). The measurement protocol included basic measurements of body mass and stretch stature and eight skinfolds with a Harpenden, Holtain, Slim Guide, and Lipowise. The ∑6 and ∑8 skinfolds and fat mass were calculated. The order in which the skinfold calipers were used was randomized. Results: No significant differences were found in either the Σ6 and Σ8 skinfolds or masses and fat percentages calculated with the skinfolds obtained with the different calipers (*p* > 0.05), and the inclusion of the covariates of sex, BMI, and hydration status of the participants showed no effect on the differences. The Bland–Altman test showed significant differences between the calipers (*p* < 0.001). Conclusion: It has been observed that the analyzed calipers have shown validity for the assessment of adiposity-related variables in a male and female sample of non-overweight, young healthy adults, but they are not interchangeable with each other when the assessment is meant to be compared over time or with other samples.

## 1. Introduction

The strong relationship between nutritional status, health, and fitness is widely known [1]. However, despite its wide use to classify nutritional status, body mass index (BMI, weight (kg)/height (m^2^)) does not provide complete information about body composition, which is imperative data for nutritional characterization [2]. Body composition assessment can provide prognostically useful data on both health and disease, providing the opportunity to monitor the effects of nutritional intervention, physical activity, and sports, as well as nutrition-related disease progression [3]. Specifically, fat mass is highly relevant in many sports, given that an excess of this component can be perceived as ‘dead weight’ when the body is resisting the forces of gravity in movements such as jumping and running [4].

Body composition can be approached on the basis of five levels of increasing complexity, in which body mass is presented as the sum of atoms, molecules, cells, tissues, and different body segments [4,5,6]. Model 1, at the atomic level, considers body mass as the sum of amount of hydrogen; carbon; oxygen; and other atoms. Model 2, at the molecular level, considers body mass as the sum of fat mass and fat-free mass, including the total body water and residual mass, which, in turn, differentiates between body mineral content, proteins, non-osseus mineral content, and glycogen. Model 3, at the cellular level, considers body mass as the sum of adipose cells, body cell mass (which includes intracellular water), other cells containing proteins, and extracellular mass (which includes extracellular water and extracellular solids). Model 4, at the tissue level, considers body mass as the sum of adipose tissue, skeletal muscular mass, and lean soft tissue, which includes muscle mass and connective tissue and residual mass. Finally, Model 5 is based on a whole-body level of complexity, in which body mass is the sum of different body segments, such as the head, trunk, and limbs [5,7,8]. In clinical and research contexts, the molecular (Model 2) and tissue (Model 4) models are largely used to assess body composition [3,4,5,8].

Despite the importance placed upon optimizing and assessing body composition, there is no universally accepted measurement method. Whilst various methods were developed to measure specific body tissues, cadaveric dissection is the only ‘direct’ method, and, in view of the impossibility of using this method on living persons, ‘indirect’ methods have been developed [4]. For example, body composition can be determined by very sophisticated methods, such as cadaver analysis, body elements measurement, neutron activation, densitometry, isotope dilution, bioelectrical impedance (BIA), dual-energy X-ray absorptiometry (DXA), computer-based tomography, and magnetic resonance imaging [9]. Although appropriate, these methods are difficult to apply in large-scale studies or routine practice, due to cost, portability, invasiveness, restraining feasibility, and/or follow up measurements, with all of them having strengths and weaknesses [4,9,10].

Anthropometric measurements, which include the weight-for-stature index, girth, length, and, particularly, skinfold thickness, have been described as reliable, low-cost, simple, highly practical, quick to be implemented and evaluated, and valid techniques for body composition assessment [10,11], combined with the fact that skinfold assessments appear to be the least affected by the factors that are difficult to control, such as food intake, hydration status, or daily activity [4]. In addition, this technique allows for the estimation of fat mass, following the molecular model (Model 2) by means of different equations [12,13,14]. Through the use of a double indirect method, from a series of skinfolds, the body density is estimated on the basis of a regression equation; from the data obtained, another formula is used to estimate fat mass [15,16]. Additionally, it allows for the estimation of tissue adipose, following the tissue model (Model 4), using an indirect mathematical method such as Kerr’s five-component model [4,17]. Therefore, by means of the anthropometric method, body mass can be segmented according to the two most popular methods used in scientific and clinical settings [3,4,5,18].

Although it is an important tool for evaluating the regional and body distribution of subcutaneous adipose tissue and body composition [5,18], the skinfold thickness results can vary widely, depending on the operators’ training and experience, as well as the measurement protocol used [3]. These protocols, such as those proposed by the ISAK [19,20], limit the scope of variation and minimize technical errors of measurement.

Furthermore, specific limitations are also found regarding the skinfold caliper operator and caliper itself [21,22]. Since adipose tissue is compressible, the pressure and time of application of the calipers needs to be standardized. [22,23,24,25]. Simultaneously, care must be taken in choosing the equipment, in order to ensure the accuracy and reproducibility of the measurements [21]. In this sense, the Harpenden caliper is the most popularly used in the scientific field [26,27], and it is considered the gold standard by expert associations in kinanthropometry, such as the ISAK [19]. Furthermore, previous studies showed its reliability and validity in estimating density by different formulas with results obtained from the hydrostatic weighing method [28], as well as for estimating body fat (with results obtained from air displacement plethysmography, although the formula used for the estimation of fat mass, once skinfolds were assessed, was not specified [27]) and BIA, with the Durnin and Womersley equations. In fact, the Harpenden caliper, through the use of the Jackson–Pollock fat percentage formula for seven and three skinfolds, had the best correlation rates against other calipers, when the assessment was performed by an experienced anthropometrist and comparing the fat percentage results with those obtained through other methods, such as hydrostatic weighing and air displacement plethysmography [29].

In recent years, digital calipers have been developed [10,11,21,30] to provide user-friendly devices and overcame the difficulty in correctly using the interval of 2–4 s measuring time [10], as well as the advantage of having a quicker and simpler reading, making this type of caliper a safe and efficient tool for assessing body composition [11]. In fact, it has been found that such digital calipers may have a lower individual predictive accuracy than traditional mechanical calipers, when compared to other methods, such as DXA or BIA [30].

Although all caliper manufacturers are supposed to follow the same rules when manufacturing these tools [25], the results of the studies that have compared different caliper models in the same population show contradictory results. In this respect, Cyrino et al. showed that two mechanical calipers, such as the Lange and Cescorf calipers, showed significantly different values when assessing different skinfolds and fat mass, according to four different equations [21]. On the other hand, other studies have shown no differences between any of the calipers, when comparing three mechanical calipers, such as the Harpenden, Lange, or Lafayette skinfold II calipers [29]. Additionally, Amaral et al. compared the measurements made with the Harpenden mechanical caliper and a new digital caliper, the Lipotool (Liposoft 2008 and Adipsmeter), with the DXA results and found that both calipers showed high agreement with each other and were equally accurate when comparing their fat mass results to those reported by the DXA method [10]. Another study showed that mechanical calipers, such as the Harpenden, Sanny, Cescorf, Lange, and Prime Vision digital calipers, obtained significantly similar data to each other when assessing fat using four different equations [11].

However, some factors could influence the agreement of the skinfolds taken with the different calipers. The skinfold reading depends on the compressibility of the adipose tissue, i.e., how the adipose tissue decreases in thickness, in reaction to the pressure exerted by the caliper [20,31,32]. This compressibility has inter- and intra-individual variations [31]; therefore, compressibility could affect skinfold measurements, thereby introducing an error in the estimation of body composition with this technique [33]. Based on cadaver studies, some factors could introduce variability in skinfold compressibility. One of them is sex, because compressibility is different between the sexes, depending on the different regions of the body, which results in the relationship between the measured skinfolds and subcutaneous adipose tissue in the measurement area being more evident in men; although, skinfold measurements gave acceptable correlation indices in both sexes [34,35]. Another one is hydration [35], as adipose tissue is 20% water [34,35], so the degree of hydration affects the thickness of the skinfolds and, consequently, its compressibility [36]. Yet another factor that could be affected is the thickness of the skinfold, which is influenced by the amount of adiposity of the subject [34,35].

However, of the previous studies that analyzed the agreement between different calipers, most only included men [11,21]; only one study included a sample of both sexes, although it did not analyze the influence of sex on the results obtained [10]. The other variables that could affect compressibility have not been analyzed in any of the studies. In fact, none of the previous articles specified the inclusion or exclusion criteria for the sample [10,11,21]. Furthermore, none of these studies included calipers such as the Holtain caliper [37,38,39,40] or Slim guide [41,42], even though these have been popularly used in scientific and clinical settings. Additionally, the Lipowise, a digital caliper model with manufacturing specificities from previous models, was not included in these previous studies—the Lipowise applies a constant pressure of 10 mol/mm^2^, offers an integrated system for skinfold measurement (which is advantageous during measurements, as it eliminates the need for the manual recording of the data), and ensures the correct use of the measurement time. This caliper is an evolution of the same equipment from another digital caliper that was previously used in different studies, the Lipotool (Liposoft 2008 and Adipsmeter) [36], but it provides improvements in some aspects, among which, we highlight the connectivity to the application via Bluetooth [32]. The Lipotool was validated in a previous study that compared the obtained results with this tool with those obtained by DXA [10].

Therefore, the purpose of this study was to investigate the agreement of four different skinfold calipers, i.e., the Harpenden, Holtain, Slim Guide, and Lipowise, and establish the differences between the sum of the skinfold and estimation of fat mass and adipose tissue using different formulae and these four calipers in a healthy young adult population.

## 2. Materials and Methods

### 2.1. Study Design and Participants

This cross-sectional study was conducted in both the Region of Murcia (Spain) and Lisbon (Portugal), with a convenience sample of 138 healthy university students, with 69 males (21.46 ± 2.52 years) and 69 females (22.19 ± 2.85 years), recruited between February and October 2021. To be considered eligible for the study, the participants had to be Caucasian, aged between 18 and 25 years old, and have a BMI between 18.5 kg m^−2^ and 24.9 kg m^−2^. They should have neither any disease that could affect body fat nor undergone hormonal or corticosteroid treatment in the three months prior to the evaluation.

Participants were excluded if, within the 24 h prior to the measurement session, they had performed vigorous physical exercise (or 12 h in case of moderate exercise), consumed products with diuretic properties, or eaten a heavy meal. Moreover, on the day of data collection, participants must not have any injury that would compromise the application of the measurement protocol, must not have performed physical exercise on the same day, and, for female participants, they must be between the 8th and 21st days of the menstrual cycle.

All the participants were volunteers and signed an informed consent form before starting the study. The study design, protocols, and procedures followed the Helsinki declaration principles and were approved by the Ethics Committees of the Faculty of Sport from the Catholic University San Antonio of Murcia (CE012109) and Faculty of Human Kinetics from the University of Lisbon (CEFMH 10/2021).

### 2.2. Procedures

For each subject, the full set of anthropometric measurements were performed in a single day, from 8 a.m. to 2 p.m., in a private room with a comfortable and standardized temperature. The measurement protocol always started with basic measurements of body mass, stretch stature, and the marking of anthropometric landmarks, followed by measurements of the skinfolds. Furthermore, the participants’ hydration status was assessed in the measurement session. Lastly, the participants were asked to provide information on basic demographics, diseases that could affect body fat, and hormonal or corticosteroid treatment.

Anthropometric variables (body mass, stretch stature, triceps, subscapular, biceps, iliac crest, supraspinale, abdominal, thigh, and calf skinfolds) were obtained, according to the guidelines of the International Society of the Advancement of Kinanthropometry (ISAK) [19], by three level 3 and two level 4 anthropometrists who were accredited by the ISAK. The mean intra-evaluator technical error of measurement (TEM) was 0.01% in the basic measurements and 1.15% in skinfolds; the mean inter-evaluator TEM was 0.04% in the basic measurements and 2.34% in skinfolds. Each set of measurements was performed twice, on the right side of the body, and registered by a recorder. A third measurement was performed on the skinfolds that obtained differences between measurements larger than 5% for skinfolds or 1% for the basic measurements. The final value for the data analysis was the mean if two measurements were taken or the median if three measurements were taken.

Body mass was measured to the nearest 0.1 kg with a digital SECA 878 scale (SECA, Hamburg, Germany), and stretch stature was measured to the nearest 0.1 cm with a portable SECA 217 stadiometer (SECA, Hamburg, Germany); both measurements were obtained with participants barefoot and wearing minimal clothes. The eight skinfolds were measured with four calibrated calipers: the Harpenden (Baty Int., UK) and Holtain calipers (Holtain, Crosswell, UK) to the nearest 0.2 mm, digital Lipowise caliper (Wisify, Porto, Portugal) to the nearest 0.1 mm, and Slim Guide caliper (Rosscraft, Canada) to the nearest 0.5 mm. Four skinfold measurement protocols (Table 1) were established, with their differentiating features being the sequence in which the four calipers were used. The application of the protocols was randomized for each participant.

Each set of skinfold measurements was taken sequentially in the order established by ISAK, and the reading was performed two seconds after the full pressure of the caliper was applied (i.e., on the 3rd s). A metronome was used to count the time between tissue compression and the reading of the skinfold value (model NW-707, Neewer, China), except for readings made with the Lipowise caliper, which uses a programmable reading time with the software Lipowise Legacy (Wisefy, Portugal). There was a pause of 5 min between the measurements of the complete skinfold profile with each caliper.

Based on the anthropometric measurements, the body mass index (BMI) (kg/m^2^), sum of six (triceps, subscapular, supraspinale, abdominal, thigh, and calf) and eight (triceps, subscapular, biceps, iliac crest, supraspinale, abdominal, thigh, and calf) skinfolds were calculated. Fat mass (%) was estimated with the equation proposed by Durnin and Womersley [14] and Faulkner [43]; tissue adipose mass was estimated by the equation proposed by Kerr [17], following the proposal from previous studies [12]. Fat mass (in kg) for all the formulae were calculated with the following equation: Fat Mass (kg) = (Fat mass (%) * Body mass (kg))/100.

To assess hydration status, researchers provided participants with sterilized containers to collect a sample of urine as close as possible to the time of measurement, which was discarded by themselves at the end of the measurement session. The urine color was determined simultaneously by two researchers in a well-lit room by placing the urine sample container next to a color chart [44]. Each color on the color chart was assigned a number, from 1 to 8, with 1 corresponding to the lightest color and 8 corresponding to the darkest color, following the codification proposal of Armstrong [44], as in previous studies [45].

### 2.3. Statistical Analysis

The normality of the distribution was verified with the Kolmogorov–Smirnov test. All the variables included in the analysis followed a normal distribution, so parametric statistical tests were performed. A descriptive analysis was performed for all the variables included. A MANCOVA test was performed to analyze the differences between the Harpenden, Holtain, Slim Guide, and Lipowise calipers, including the covariates sex, BMI, and hydration status, in order to study their influence on the possible differences. The software used to perform the normality and MANCOVA tests was SPSS (v.23, IBM, USA). Agreement between calipers was determined using Lin’s concordance correlation coefficient (CCC), including precision (ρ) and accuracy (Cb) indexes, as well as by McBride’s strength concordance (almost perfect > 0.99; substantial > 0.95 to 0.99; moderate = 0.90–0.95; and poor < 0.90) [46], following previous research [47]. Pearson’s correlation and Bland–Altman tests were used to determine the agreement and interchangeability between the different calipers, with respect to the Harpenden caliper. For Pearson’s correlation, the following ranges were established: r < 0.5 for low correlation, 0.5–0.7 for moderate correlation, and >0.7 for high correlation [48]. The software used to perform Lin’s concordance correlation, Pearson’s correlations, and the Bland–Altman test was MedCalc Statistical Software v.20.106 (Mariakerke, Belgium)). The level of significance was set at *p* ≤ 0.05.

## 3. Results

The descriptive statistics of the participants can be observed in Table 2.

In general, the MANCOVA results did not show significant differences in the Σ6 and Σ8 skinfolds, masses, or fat percentages with the different formulae calculated and the skinfolds obtained with the different calipers (Table 3). The inclusion of the covariates sex, BMI, and hydration status of the participants showed no effects on the differences between the skinfold calipers (Table 3).

Table 4 shows the concordance between the four calipers analyzed. A moderate to almost perfect concordance was observed in all the measurements and calculated variables.

Table 5 shows a substantial significant correlation between all the calipers respect Harpenden for all the variables and the confidence intervals and Bland–Altman 95% limits of agreement between methods. However, when compared with the results obtained with the Harpenden caliper, significant differences were observed between all calipers in most of the variables.

**Figure 1:**
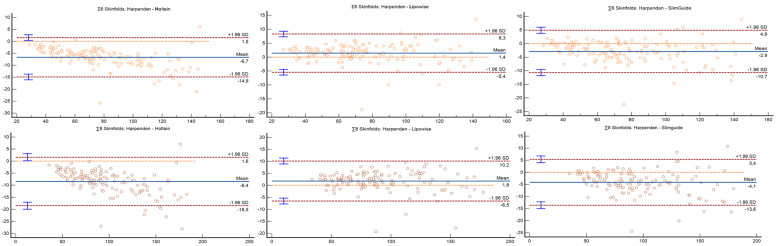
Bland–Altman plot for the 6 and 8 skinfolds.

**Figure 2:**
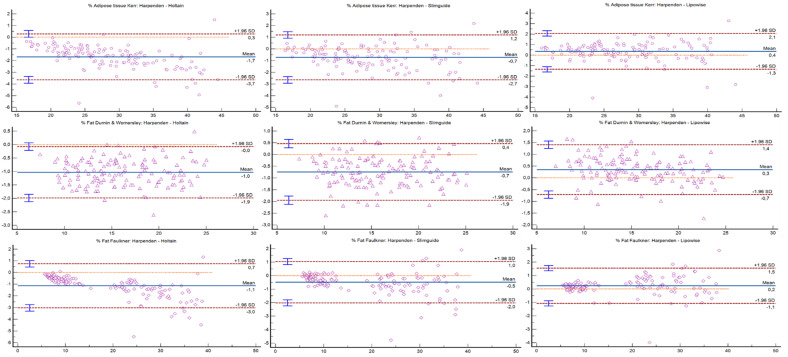
Bland–Altman plot for the percentage of adipose tissue and fat mass formulas.

**Figure 3:**
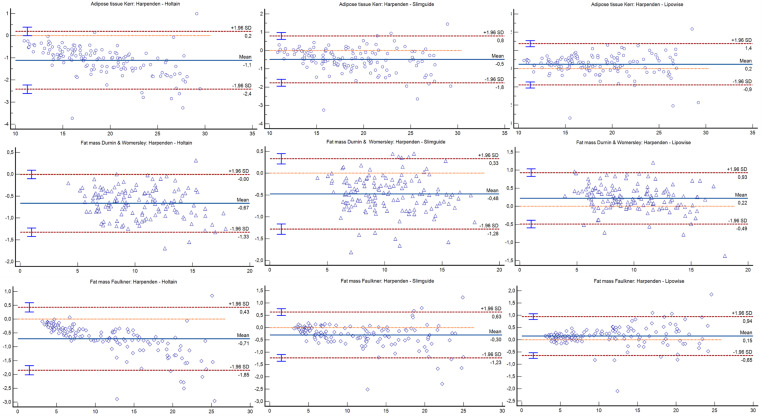
Bland–Altman plot for the kilograms of adipose tissue and fat mass formulas.

Bland–Altman plots can be observed in Figure 1, Figure 2 and Figure 3. The Holtain and Slim Guide calipers overestimated skinfolds, while the Lipowise slightly underestimated skinfolds, as compared to the Harpenden caliper. The figures show that the higher the percentage of fat, the greater the disagreement between calipers.

## 4. Discussion

The aim of the present study was to analyze the agreement of four different skinfold calipers and establish the differences between the sum of the skinfold and estimation of the fat mass and adipose tissue using different formulae and four calipers in a healthy young adult population. In this sense, the main finding of the present work was that no differences were found between the values measured with the four calipers in the eight individual skinfolds, and no differences were observed in the calculated mass and fat percentage either, showing a high degree of agreement among all the calipers analyzed. This could be due to the fact that the skinfold calipers are constructed with similar technical specifications, in terms of the pressure they exert on the subcutaneous tissue [22]. It has been observed that the pressure exerted by the skinfold caliper has a significant effect on both the measured skinfold thickness and reproducibility of that measurement [23]. In this regard, average pressures of 10.00 g·mm^2^ on the ascending scale and 8.25 g·mm^2^ on the descending scale have been recommended, so as to not compromise the reproducibility of the measurements [23,24,25]. In addition, a pressure difference over a range between 2 and 40 mm of opening in the skinfold caliper branches of 0.5–2 g·mm^2^, depending on the model used, is considered acceptable for reducing the effect of skin hysteresis [22,23]. Despite these recommendations, when the technical characteristics of different skinfold calipers have been analyzed, it has been observed that the pressures measured are slightly below the values specified by the manufacturers, without compromising the validity and reliability of the skinfold calipers, since the differences are within the range that is considered acceptable [25].

The differences found in previous studies in the pressure values of the different skinfold caliper models could explain why the Bland–Altman test indicated that the skinfold calipers used in the present work were not interchangeable. The Harpenden caliper is the most traditionally used skinfold caliper to measure subcutaneous fat, showing validity and reliability, with respect to other techniques used [49,50]. Another skinfold caliper that has been classically used for the assessment of body composition is the Holtain caliper [51], as it complies with the internationally established construction standards. The Slim Guide has also been validated against other skinfold calipers [52], and it meets the construction specifications of those mentioned above. Recently, the Lipowise caliper emerged, which was built following the accepted indications, in terms of construction characteristics. Taking the Harpenden skinfold caliper as a reference, as it has been the most widely used in research [12,53], the Lipowise caliper comes closest to the values reported by the former, finding that it slightly underestimated the skinfolds. In the case of the Holtain and Slim Guide skinfold calipers, it was observed that they overestimated the results, with respect to the Harpenden one, with similar values between them. These results are in agreement with those observed in previous studies, which analyzed the agreement and interchangeability of the different methods for estimating body composition, such as dual energy X-ray absorptiometry, air displacement plethysmography, electrical bioimpedance, and even the use of different devices within the same method, finding that they all have reliability and internal validity, but that it is not possible to compare the data obtained with different methods or different devices within the same method, so they are not interchangeable with each other [27,54,55].

In relation to BMI, it has been observed that, in populations with a higher BMI, the error that occurs when taking skinfolds increases, due, in part, to the compressibility of the subcutaneous adipose tissue and lower pressure exerted by the skinfold calipers in the extreme ranges of the opening [22,56]. Similar problems have been found in underweight individuals, as the higher pressure exerted by the skinfold calipers in the first degrees of opening, together with the acceptable margin of error for skinfold measurements (set at 5% of the assessed value), causes the reproducibility of the method to decrease [19,22,25]. That is why, in order to try to minimize the error introduced in the measurements, BMI was established as the inclusion criterion. However, in spite of this, to control the effect of BMI on the possible differences between the skinfold calipers analyzed, it was introduced as a covariate in the statistical analysis, and it was found that it had no influence on the results shown. However, it was observed in the Bland–Altman plots that the higher the percentage of fat, the greater the disagreement between calipers. Therefore, the influence that the amount of adipose tissue might have on the degree of agreement shown between calipers is an issue that needs to be addressed in future studies, when assessing populations with large amounts of adiposity, such as overweight or obese individuals.

Similarly, it has been observed that the hydration status of the subject at the time of the assessment can affect the results obtained, in terms of body composition [57]. However, in the present study, the hydration status did not have an effect on the differences found between the skinfold calipers analyzed. These results are in agreement with what has been observed in previous studies, in which skinfolds were found to have little susceptibility to changes in hydration status [58]. However, further research should repeat this study in other populations, with different controlled hydration protocols.

Previous studies have observed differences between the male and female populations, in terms of the percentage and distribution of fat mass [59,60]. In the case of the female population, it has been observed that there is a tendency to have a higher percentage of fat, as well as to accumulate it as subcutaneous fat in the region of the hips and lower limbs, known as the gynecoid prototype [59]. However, in the case of the male population, the storage of fat mass occurs to a greater extent in the abdominal area, with more visceral fat, known as the android prototype [59]. Despite the clear evidence of differences in fat mass distribution and storage between men and women, when the sex covariate was introduced in the present analysis, only differences between the skinfold calipers in the triceps and biceps skinfolds were observed, which could be due to the unequal distribution of adipose tissue between sexes. On the other hand, previous studies have found differences between men and women in the skinfold variability measured with the same skinfold caliper, which was attributed to differences in subcutaneous adipose tissue compressibility between sexes [61]. If true, this source of variability would affect the measurements made with all the skinfold calipers in the present study and explain the absence of differences in most of the variables analyzed when including the sex covariate. However, since there are no studies that have verified these differences using different skinfold calipers validated in male and female populations of different ages, future studies should corroborate the findings of the present study.

The present investigation is not without limitations. Among them, it should be noted that, although the measurers who took the data were ISAK level 3 and 4 accredited kinanthropometrists with a low TEM and the variables were measured repeatedly to avoid random error, the measurers could be a source of error, with respect to the final result. Nevertheless, when it comes to analyzing the validity of different skinfold calipers in the field, there is no alternative to the protocol used in the present investigation.

## 5. Conclusions

In the present study, it was observed that the Harpenden, Holtain, Slim Guide, and Lipowise skinfold calipers showed similar values for the assessment of the variables related to adiposity in a male and female sample of young adults who were not overweight, with a high agreement between all of them. However, it has also been observed that these skinfold calipers are not interchangeable with each other, so that, within the practical implications derived from this study, it would be advisable, whenever possible, to perform the measurements with the same model of skinfold caliper when we intend to perform a follow-up of an individual or compare the results measured with one or several studies. However, if it is not possible to perform the measurements with the same skinfold caliper, the skinfold calipers that yielded the most similar values were the Harpenden and Lipowise caliper, as well as the Holtain and Slim Guide calipers, respectively.

## Figures and Tables

**Table 1:** Skinfold measurement protocols.

Protocol 1	Protocol 2	Protocol 3	Protocol 4
Harpenden	Holtain	Slim Guide	Lipowise
Holtain	Slim Guide	Lipowise	Harpenden
Slim Guide	Lipowise	Harpenden	Holtain
Lipowise	Harpenden	Holtain	Slim Guide

**Table 2:** Descriptive statistics of the participants.

Variable	Mean ± SD	
	Men (n = 69)	Women (n = 69)
Age (years old)	21.46 ± 2.52	22.19 ± 2.85
Body mass (kg)	68.73 ± 8.03	59.50 ± 6.12
Height (cm)	175.67 ± 6.73	164.91 ± 6.14
BMI (kg/m^2^)	22.21 ± 1.61	21.85 ± 1.71
Hydration status (score from 1 to 8)	5.88 ± 1.46	5.33 ± 1.75

**Table 3:** Descriptive statistics and differences between the variables calculated with different calipers.

Variable	MEAN ± SD				ANOVA			ANCOVA								
								Calipers * Sex			Calipers * BMI			Calipers * Hydration		
	Lipowise	Harpenden	Holtain	Slim Guide	F	*p*	Eta	F	*p*	Eta	F	*p*	Eta	F	*p*	Eta
Triceps sf (mm)	12.69 ± 5.75	12.97 ± 5.82	14.07 ± 6.21	13.72 ± 6.11	0.968	0.327	0.007	7.725	0.006	0.055	0.814	0.368	0.006	0.568	0.452	0.004
Subscapular sf (mm)	9.56 ± 3.28	9.84 ± 3.26	10.58 ± 3.60	10.44 ± 3.66	0.024	0.877	0.000	0.000	0.984	0.000	0.000	0.997	0.000	3.517	0.063	0.026
Biceps sf (mm)	5.07 ± 3.03	5.33 ± 2.77	5.98 ± 3.24	5.79 ± 3.10	2.338	0.129	0.017	4.009	0.047	0.029	4.394	0.038	0.032	0.354	0.553	0.003
Iliac crest sf (mm)	12.05 ± 5.24	12.19 ± 5.22	13.30 ± 5.47	12.95 ± 5.27	3.833	0.052	0.028	0.000	0.994	0.000	2.775	0.098	0.020	0.083	0.773	0.001
Supraspinale sf (mm)	8.08 ± 3.78	8.28 ± 3.71	9.07 ± 4.05	8.76 ± 3.88	0.864	0.354	0.006	1.107	0.295	0.008	2.090	0.151	0.015	1.797	0.182	0.013
Abdominal sf (mm)	14.77 ± 6.94	14.85 ± 6.70	16.21 ± 7.20	14.97 ± 6.65	0.319	0.573	0.002	0.246	0.621	0.002	0.461	0.498	0.003	0.000	0.987	0.000
Thigh sf (mm)	17.70 ± 8.16	17.95 ± 8.33	19.56 ± 8.80	18.42 ± 8.34	0.048	0.827	0.000	0.203	0.653	0.002	0.225	0.636	0.002	0.001	0.981	0.000
Calf sf (mm)	10.45 ± 5.67	10.76 ± 5.65	11.84 ± 6.17	11.23 ± 5.83	1.888	0.172	0.014	2.148	0.145	0.016	2.561	0.112	0.019	0.483	0.488	0.004
Σ6 skinfolds (mm)	73.24 ± 28.71	74.66 ± 28.55	81.33 ± 30.77	77.53 ± 29.42	0.125	0.724	0.001	0.967	0.327	0.007	0.319	0.573	0.002	0.259	0.612	0.002
Σ8 skinfolds (mm)	90.36 ± 35.46	92.17 ± 35.08	100.61 ± 37.87	96.27 ± 36.21	0.032	0.858	0.000	0.675	0.413	0.005	0.008	0.928	0.000	0.436	0.510	0.003
AT Kerr (%)	27.59 ± 6.86	27.95 ± 6.81	29.63 ± 7.34	28.68 ± 7.04	1.420	0.236	0.010	2.216	0.139	0.016	1.592	0.209	0.012	2.206	0.140	0.016
AT Kerr (Kg)	18.29 ± 4.55	18.53 ± 4.52	19.64 ± 4.87	19.01 ± 4.67	1.420	0.236	0.010	2.216	0.139	0.016	1.592	0.209	0.012	2.206	0.140	0.016
FM Durnin and Womersley (%)	15.85 ± 5.41	16.23 ± 5.25	17.36 ± 5.32	17.06 ± 5.30	0.064	0.801	0.000	0.107	0.744	0.001	2.103	0.149	0.015	0.712	0.400	0.005
FM Durnin and Womersley (Kg)	10.51 ± 3.59	10.76 ± 3.49	11.51 ± 3.52	10.91 ± 3.55	1.172	0.281	0.009	1.362	0.245	0.010	0.043	0.837	0.000	0.811	0.369	0.006
FM Faulkner (%)	12.68 ± 2.65	12.81 ± 2.59	13.42 ± 2.81	13.11 ± 2.72	0.177	0.674	0.001	0.88′	0.350	0.007	0.048	0.826	0.000	0.093	0.761	0.001
FM Faulkner (Kg)	8.24 ± 2.05	8.22 ± 2.02	8.62 ± 2.16	8.41 ± 2.11	0.757	0.386	0.006	0.350	0.555	0.003	0.681	0.411	0.005	0.078	0.781	0.001

Abbreviations: sf, skinfold; AT, adipose tissue; FM, fat mass. *: Covariate included in the ANCOVA test.

**Table 4:** Lin’s concordance correlation coefficient between the different calipers in the analyzed variables.

Calipers	Variable	Lin’s Concordance Correlation Coefficient		
		CCC	ρ	C_b_
Harpenden-Holtain	Triceps skinfold	0.975	0.993	0.982
	Subscapular skinfold	0.962	0.989	0.972
	Biceps skinfold	0.946	0.980	0.965
	Iliac crest skinfold	0.966	0.988	0.978
	Supraspinale skinfold	0.963	0.987	0.976
	Abdominal skinfold	0.959	0.980	0.979
	Thigh skinfold	0.956	0.974	0.981
	Calf skinfold	0.972	0.992	0.980
	Σ6 skinfolds	0.966	0.993	0.973
	Σ8 skinfolds	0.964	0.993	0.971
	Adipose tissue Kerr (%)	0.963	0.993	0.970
	Adipose tissue Kerr (Kg)	0.963	0.993	0.970
	Fat mass Durnin and Womersley (%)	0.972	0.994	0.978
	Fat mass Durnin and Womersley (Kg)	0.972	0.994	0.978
	Fat mass Faulkner (%)	0.963	0.991	0.972
	Fat mass Faulkner (Kg)	0.974	0.994	0.980
Harpenden-Slimguide	Triceps skinfold	0.982	0.991	0.991
	Subscapular skinfold	0.961	0.982	0.979
	Biceps skinfold	0.964	0.972	0.992
	Iliac crest skinfold	0.971	0.982	0.989
	Supraspinale skinfold	0.974	0.983	0.990
	Abdominal skinfold	0.982	0.982	1.000
	Thigh skinfold	0.965	0.966	0.998
	Calf skinfold	0.984	0.988	0.996
	Σ6 skinfolds	0.986	0.991	0.995
	Σ8 skinfolds	0.984	0.991	0.993
	Adipose tissue Kerr (%)	0.985	0.991	0.994
	Adipose tissue Kerr (Kg)	0.985	0.991	0.994
	Fat mass Durnin and Womersley (%)	0.976	0.990	0.986
	Fat mass Durnin and Womersley (Kg)	0.980	0.991	0.988
	Fat mass Faulkner (%)	0.996	0.998	0.999
	Fat mass Faulkner (Kg)	0.996	0.997	0.999
Harpenden-Lipowise	Triceps skinfold	0.989	0.991	0.999
	Subscapular skinfold	0.981	0.985	0.996
	Biceps skinfold	0.964	0.972	0.992
	Iliac crest skinfold	0.989	0.989	1.000
	Supraspinale skinfold	0.986	0.988	0.998
	Abdominal skinfold	0.978	0.979	0.999
	Thigh skinfold	0.970	0.971	0.999
	Calf skinfold	0.989	0.990	0.998
	Σ6 skinfolds	0.991	0.993	0.999
	Σ8 skinfolds	0.991	0.993	0.999
	Adipose tissue Kerr (%)	0.991	0.992	0.999
	Adipose tissue Kerr (Kg)	0.991	0.992	0.999
	Fat mass Durnin and Womersley (%)	0.989	0.993	0.996
	Fat mass Durnin and Womersley (Kg)	0.991	0.994	0.997
	Fat mass Faulkner (%)	0.998	0.998	1.000
	Fat mass Faulkner (Kg)	0.998	0.998	1.000
Holtain-Slimguide	Triceps skinfold	0.988	0.990	0.998
	Subscapular skinfold	0.978	0.979	0.999
	Biceps skinfold	0.976	0.979	0.997
	Iliac crest skinfold	0.981	0.984	0.997
	Supraspinale skinfold	0.980	0.984	0.996
	Abdominal skinfold	0.960	0.979	0.981
	Thigh skinfold	0.981	0.991	0.990
	Calf skinfold	0.984	0.990	0.993
	Σ6 skinfolds	0.985	0.994	0.991
	Σ8 skinfolds	0.986	0.994	0.992
	Adipose tissue Kerr (%)	0.983	0.993	0.990
	Adipose tissue Kerr (Kg)	0.983	0.993	0.990
	Fat mass Durnin and Womersley (%)	0.988	0.991	0.998
	Fat mass Durnin and Womersley (Kg)	0.990	0.992	0.998
	Fat mass Faulkner (%)	0.996	0.998	0.998
	Fat mass Faulkner (Kg)	0.995	0.998	0.997
Holtain-Lipowise	Triceps skinfold	0.960	0.988	0.971
	Subscapular skinfold	0.935	0.981	0.953
	Biceps skinfold	0.940	0.982	0.957
	Iliac crest skinfold	0.959	0.986	0.973
	Supraspinale skinfold	0.952	0.986	0.966
	Abdominal skinfold	0.962	0.983	0.979
	Thigh skinfold	0.967	0.993	0.974
	Calf skinfold	0.962	0.992	0.970
	Σ6 skinfolds	0.957	0.995	0.962
	Σ8 skinfolds	0.955	0.994	0.960
	Adipose tissue Kerr (%)	0.952	0.994	0.958
	Adipose tissue Kerr (Kg)	0.952	0.994	0.958
	Fat mass Durnin and Womersley (%)	0.945	0.992	0.953
	Fat mass Durnin and Womersley (Kg)	0.954	0.992	0.962
	Fat mass Faulkner (%)	0.988	0.999	0.989
	Fat mass Faulkner (Kg)	0.987	0.999	0.988
Slimguide-Lipowise	Triceps skinfold	0.971	0.988	0.983
	Subscapular skinfold	0.937	0.973	0.963
	Biceps skinfold	0.953	0.980	0.972
	Iliac crest skinfold	0.969	0.984	0.985
	Supraspinale skinfold	0.967	0.983	0.984
	Abdominal skinfold	0.981	0.982	0.999
	Thigh skinfold	0.987	0.991	0.996
	Calf skinfold	0.982	0.991	0.990
	Σ6 skinfolds	0.983	0.994	0.989
	Σ8 skinfolds	0.980	0.994	0.986
	Adipose tissue Kerr (%)	0.980	0.993	0.988
	Adipose tissue Kerr (Kg)	0.980	0.993	0.988
	Fat mass Durnin and Womersley (%)	0.961	0.991	0.970
	Fat mass Durnin and Womersley (Kg)	0.967	0.991	0.976
	Fat mass Faulkner (%)	0.996	0.999	0.997
	Fat mass Faulkner (Kg)	0.995	0.998	0.997

Abbreviations: CCC, concordance correlation coefficient; ρ, precision; C_b_, accuracy.

**Table 5:** Differences between calipers, with respect to the Harpenden caliper and Bland–Altman limits of agreement.

Caliper	Pearson’s *r* (*p*)	Harpenden—Caliper		
		Mean Diff (95% CI)	95% Limits of Agreement	*p*
Triceps skinfold				
Holtain	r = 0.99; *p* < 0.001	−0.74 (−0.93 to −0.55)	−2.71 to 0.53	<0.000
Slim Guide	r = 0.99; *p* < 0.001	−1.09 (−1.27 to −0.92)	−1.27 to 1.84	<0.000
Lipowise	r = 0.99; *p* < 0.001	0.29 (0.11 to 0.47)	−2.44 to 0.96	<0.000
Subscapula skinfold				
Holtain	r = 0.98; *p* < 0.001	−0.47 (−0.64 to −0.30)	−1.94 to 0.47	<0.000
Slim Guide	r = 0.98; *p* < 0.001	−0.65 (−0.82 to −0.49)	−0.84 to 1.42	<0.000
Lipowise	r = 0.98; *p* < 0.001	0.26 (0.10 to 0.42)	−2.11 to 0.92	<0.000
Biceps skinfold				
Holtain	r = 0.98; *p* < 0.001	−0.47 (−0.63 to −0.30)	−2.14 to 0.83	<0.000
Slim Guide	r = 0.98; *p* < 0.001	−0.65 (−0.81 to −0.49)	−1.17 to 1.69	0.007
Lipowise	r = 0.97; *p* < 0.001	0.26 (0.10 to 0.42)	−1.94 to 1.01	<0.000
Iliac crest skinfold				
Holtain	r = 0.99; *p* < 0.001	−0.77 (−1.00 to −0.54)	−2.80 to 0.58	<0.000
Slim Guide	r = 0.99; *p* < 0.001	−1.11 (−1.30 to −0.92)	−1.39 to 1.65	<0.000
Lipowise	r = 0.98; *p* < 0.001	0.13 (−0.05 to 0.31)	−2.73 to 1.20	0.288
Supraspinale skinfold				
Holtain	r = 0.99; *p* < 0.001	−0.48 (−0.64 to −0.31)	−2.20 to 0.60	<0.000
Slim Guide	r = 0.99; *p* < 0.001	−0.80 (−0.96 to −0.64)	−0.95 to 1.35	<0.000
Lipowise	r = 0.98; *p* < 0.001	0.20 (0.06 to 0.33)	−1.90 to 0.94	0.001
Abdominal skinfold				
Holtain	r = 0.98; *p* < 0.001	−0.12 (−0.41 to 0.18)	−4.27 to 1.55	1.000
Slim Guide	r = 0.98; *p* < 0.001	−1.36 (−1.70 to −1.02)	−18.18 to 12.47	<0.000
Lipowise	r = 0.98; *p* < 0.001	0.08 (−0.25 to 0.41)	−2.62 to 2.38	1.000
Thigh skinfold				
Holtain	r = 0.97; *p* < 0.001	−0.47 (−0.97 to 0.03)	−5.54 to 2.33	0.076
Slim Guide	r = 0.97; *p* < 0.001	−1.61 (−2.05 to −1.16)	−3.69 to 4.19	<0.000
Lipowise	r = 0.96; *p* < 0.001	0.25 (−0.21 to 0.71)	−4.72 to 3.79	0.909
Calf skinfold				
Holtain	r = 0.99; *p* < 0.001	−0.47 (−0.68 to −0.26)	−2.85 to 0.70	<0.000
Slim Guide	r = 0.99; *p* < 0.001	−1.07 (−1.26 to −0.89)	−1.24 to 1.87	<0.000
Lipowise	r = 0.99; *p* < 0.001	0.32 (0.14 to 0.50)	−2.26 to 1.33	<0.000
Σ6 skinfolds				
Holtain	r = 0.99; *p* < 0.001	−6.67 (−7.38 to −5.97)	−14.88 to 1.54	<0.001
Slim Guide	r = 0.99; *p* < 0.001	−2.87 (−3.54 to −2.20)	−10.65 to 4.91	<0.001
Lipowise	r = 0.99; *p* < 0.001	1.42 (0.83 to 2.01)	−5.44 to 8.28	<0.001
Σ8 skinfolds				
Holtain	r = 0.99; *p* < 0.001	−8.44 (−9.30 to −7.57)	−18.49 to 1.62	<0.001
Slim Guide	r = 0.99; *p* < 0.001	−4.10 (−4.92 to −3.29)	−13.60 to 5.39	<0.001
Lipowise	r = 0.99; *p* < 0.001	1.81 (1.10 to 2.53)	−6.54 to 10.16	<0.001
Adipose tissue Kerr (%)				
Holtain	r = 0.99; *p* < 0.001	−1.68 (−1.85 to −1.51)	−3.65 to 0.29	<0.001
Slim Guide	r = 0.99; *p* < 0.001	−0.73 (−0.89 to −0.56)	−2.65 to 1.20	<0.001
Lipowise	r = 0.99; *p* < 0.001	0.36 (0.21 to 0.51)	−1.35 to 2.07	<0.001
Adipose tissue Kerr (Kg)				
Holtain	r = 0.99; *p* < 0.001	−1.11 (−1.23 to −1.00)	−2.42 to 0.19	<0.001
Slim Guide	r = 0.99; *p* < 0.001	−0.48 (−0.59 to −0.37)	−1.76 to 0.79	<0.001
Lipowise	r = 0.99; *p* < 0.001	0.24 (0.14 to 0.34)	−0.89 to 1.37	<0.001
Fat mass Durnin and Womersley (%)				
Holtain	r = 0.99; *p* < 0.001	−1.03 (−1.11 to −0.95)	−1.99 to −0.07	<0.001
Slim Guide	r = 0.99; *p* < 0.001	−0.74 (−0.84 to −0.64	−1.95 to 0.46	<0.001
Lipowise	r = 0.99; *p* < 0.001	0.35 (0.26 to 0.44)	−0.71 to 1.41	<0.001
Fat mass Durnin and Womersley (Kg)				
Holtain	r = 0.99; *p* < 0.001	−0.67 (−0.72 to −0.61)	−1.32 to −0.01	<0.001
Slim Guide	r = 0.99; *p* < 0.001	−0.47 (−0.54 to −0.41)	−1.28 to 0.33	<0.001
Lipowise	r = 0.99; *p* < 0.001	0.22 (0.16 to 0.28)	−0.49 to 0.93	<0.001
Fat mass Faulkner (%)				
Holtain	r = 0.99; *p* < 0.001	−1.14 (−1.30 to −0.98)	−3.02 to 0.75	<0.001
Slim Guide	r = 0.99; *p* < 0.001	−0.48 (−0.62 to −0.35)	−2.01 to 1.04	<0.001
Lipowise	r = 0.99; *p* < 0.001	0.23 (0.12 to 0.35)	−1.07 to 1.54	<0.001
Fat mass Faulkner (Kg)				
Holtain	r = 0.99; *p* < 0.001	−0.71 (−0.81 to −0.61)	−1.85 to 0.42	<0.001
Slim Guide	r = 0.99; *p* < 0.001	−0.30 (−0.38 to −0.22)	−1.23 to 0.63	<0.001
Lipowise	r = 0.99; *p* < 0.001	0.15 (0.08 to 0.22)	−0.65 to 0.94	<0.001

## Data Availability

The data of the present research are available from the corresponding author on reasonable request.
